# Methyprednisolone i.v. alters levels of CGRP and melatonin in cluster headache patients

**DOI:** 10.1186/1129-2377-14-S1-P63

**Published:** 2013-02-21

**Authors:** L Neeb, L Anders, P Euskirchen, J Hoffmann, U Reuter

**Affiliations:** 1 Charite Universitaetsmedizin Berlin, Germany

## Introduction

Treatment with steroids for short term cluster headache (CH) prophylaxis is a widely accepted therapy. However, the mechanism of action of steroids in CH prophylaxis is unknown. Various studies could show that the trigeminovascular system and the hypothalamus play probably a key role in CH pathophysiology. The neuropeptid calcitonin gene related peptide (CGRP) is released in an acute attack indicating activation of the trigeminovasclular system.[1] The hypothalamus regulates the circadian secretion of melatonin which is reduced in CH patients during a bout.[2]

## Objective

The aim of this study was to assess if treatment with high dose methylprednisolone (MP) i.v. inhibits release of CGRP and influences secretion of melatonin in CH.

## Methods

10 patients with episodic Cluster headache and 5 control patients with an acute episode of multiple sclerosis (MS) who should receive MP i.v. were included in the study. Patients were treated at the beginning of an episode (CH or MS) with a course of once daily 1g MP i.v. for three days followed by oral tapering. CGRP was assessed in plasma of the external jugular vein and the metabolite of melatonin in urine - 6-sulfatoxymelatonin – was collected separately during the day and night. Measurements were done before as well as one day, one and two weeks after start of treatment. Patients recorded the number and severity of headache attacks each day.

## Results

Treatment with MP led to a transient and significant decline of headache frequency. Simultaneously, CGRP plasma levels were reduced up to one week after end of treatment. Secretion of melatonin increased one and two weeks after treatment significantly. No significant changes could be observed in the control group.

## Conclusion

The results could point to a possible mechanism of action of steroids in cluster headache prophylaxis. The altered secretion pattern could be explained through a direct effect of MP on the trigeminovascular system and the hypothalamus but could also be a consequence of the reduced frequency of attacks.
